# Controlled Experimental Infection in Pigs with a Strain of Yersinia enterocolitica Harboring Genetic Markers for Human Pathogenicity: Colonization and Stability

**DOI:** 10.1128/iai.00157-23

**Published:** 2023-05-31

**Authors:** Emilie Esnault, Alicia Rouaud, Annie Labbé, Catherine Houdayer, Yann Bailly, Emmanuelle Houard, Stéphanie Bougeard, Frédéric Paboeuf, Nicolas Eterradossi, Marianne Chemaly, Martine Denis

**Affiliations:** a Unit for Hygiene of Poultry and Pork Products, ANSES, Laboratory of Ploufragan-Plouzané-Niort, Ploufragan, France; b Unit for SPF Pig Production and Experimentation, ANSES, Laboratory of Ploufragan-Plouzané-Niort, Ploufragan, France; c Unit for Epidemiology, Health and Welfare Research, ANSES, Laboratory of Ploufragan-Plouzané-Niort, Ploufragan, France; d ANSES, Laboratory of Ploufragan-Plouzané-Niort, Ploufragan, France; University of Pennsylvania

**Keywords:** *Yersinia enterocolitica*, pigs, ELISA, MLVA, virulence genes, similar to animal models, genetic stability, animal models, pulsed-field gel electrophoresis

## Abstract

Yersinia enterocolitica (*Ye*) is one of the major causes of foodborne zoonosis. The BT4/O:3 bioserotype is most commonly isolated in human infections. Pigs are considered the main reservoir of *Ye*, and hence, understanding the dynamics of infection by this pathogen at the individual and group levels is crucial. In the present study, an experimental model was validated in Large White pigs infected with a BT4/O:3 strain. This study showed that *Ye* contamination in pigs may occur via the introduction of the bacteria not only by mouth but also by snout, with a colonization process consisting of three periods corresponding to three contamination statuses of pigs: P1, corresponding to the 24 h following ingestion or inhalation of *Ye* with the appearance of bacteria in tonsils or in feces; P2, from 2 days postinoculation (dpi), corresponding to expansion of *Ye* and colonization of the digestive system and extraintestinal organs associated with an IgG serological response; and P3, after 21 dpi, corresponding to regression of colonization with intermittent *Ye* detection in tonsils and feces. Although the inoculated strain persisted up to 56 dpi in all pigs, genetic variations with the loss of the gene *yadA* (a gene involved in human infection) and the emergence of two new multilocus variable-number tandem-repeat analysis (MLVA) profiles were observed in 33% of the 30 isolates studied. This experimental infection model of pigs by *Ye* provides new insights into the colonization steps in pigs in terms of bacterial distribution over time and bacterial genetic stability.

## INTRODUCTION

Pigs are considered the main reservoir of Yersinia enterocolitica (*Ye*), a zoonotic agent ranking third among the causes of human enteritis in Europe in 2020 (1, 2).

The species *Ye* is divided into six biotypes, regardless of the pathogenicity of the strain in humans (3, 4). In addition to biotypes, more than 50 serotypes have been defined based on *Yersinia* polysaccharide O antigens (4). To induce yersiniosis, a set of virulence factors related to plasmid pYV and to chromosomal genes must be present, which contribute to host colonization and prevent the action of specific and nonspecific host defense mechanisms (1).

In Europe and in numerous other countries, human-pathogenic strains include biotype 4 (BT4), BT3, and BT2. The predominant biotypes may vary by geographic region, but in Europe, BT4 remains the most common biotype isolated in human cases, with a frequency of 88% (2). Despite some geographic variation, BT4 (mostly comprising serotype O:3 strains) is also the biotype most frequently isolated from pigs and may represent 65% to 100% of the biotypes isolated from this species. BT3 and BT2 are also found on pig tonsils and in feces at slaughterhouses, but in general less frequently (5–9). Pigs are therefore considered the main reservoir of human-pathogenic strains. Genotypic similarity of *Ye* strains isolated from human cases and infected pigs has been demonstrated using pulsed-field gel electrophoresis (PFGE) and multilocus variable-number tandem-repeat (VNTR) analysis (MLVA) (10, 11). Nevertheless, the genetic stability of the strains and the accuracy of these typing methods are still in question (12).

Carriage of *Ye* by pigs is usually without clinical repercussions or visible lesions, which prevents its detection at the farm level but also at the slaughterhouse during ante- and postmortem inspections (13). Hence, understanding the ecology of this foodborne pathogen at the farm level is crucial, as its prevalence varies and may depend on factors such as age and farm management (14–16).

Although the contamination route (oral or nasal, considering pig burrowing behavior) has not been fully elucidated, studies in animal husbandry and animal experiments have found that contamination likely occurs by ingestion of the bacteria through contaminated food, material, or water and occurs mainly during the fattening period (9, 13–15).

The ingested bacteria colonize the digestive tract and may invade the body via the M cells of Peyer’s patches in the terminal ileum (17). Importantly, *Ye* strains harbor different virulence factors that promote adhesion, invasion of phagocytic cells, or resistance to intracellular or extracellular (18, 19) antibacterial defense mechanisms. Among these factors, the plasmid-encoded YadA and chromosome-encoded Ail proteins are commonly used as virulence markers (20, 21). As a result of intestinal colonization, piglets start shedding the bacteria in feces around the age of 4 to 12 weeks, with maximum excretion at around 8 to 20 weeks of age. Fecal prevalence tends to decrease thereafter, although antibodies remain detectable until slaughter (13, 22). The aim of this study was to propose an experimental *Ye* infection model in pigs and to determine the influence of the infection route (oral versus nasal) on symptoms, carriage, shedding, and occurrence of the bacteria in organs over time. The impact of colonization on the genetic stability of the strain was also examined.

## RESULTS

### Controlled oral and nasal *Ye* inoculations reproduce symptomless Ye infection as it occurs under farm conditions.

**FIG 1:**
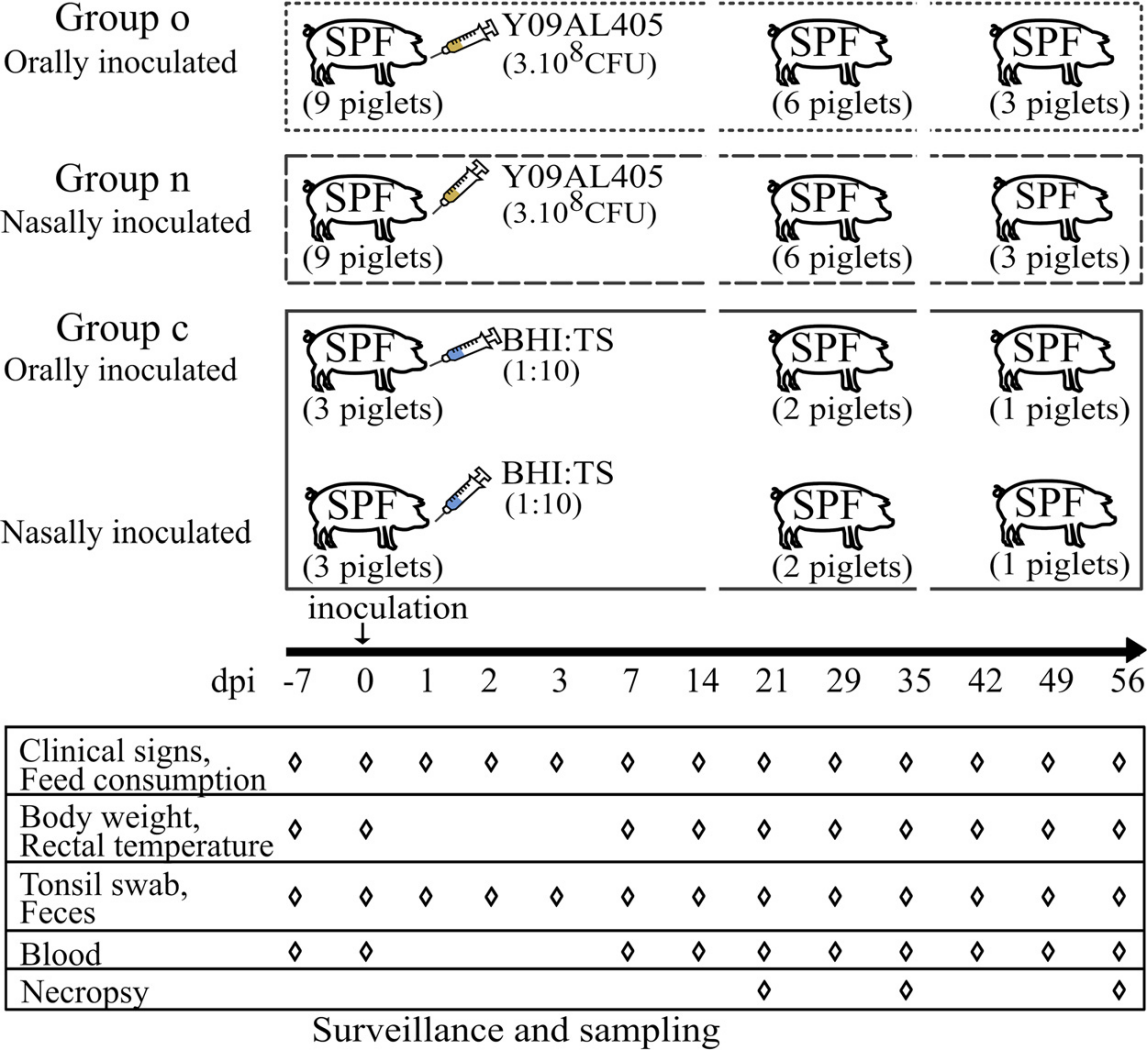
Experimental scheme of this study. The 18 piglets included in this study were distributed in three groups in separate confined animal houses. Water and feed were available *ad libitum* during the whole experimental period. Pigs were inoculated at 8 weeks of age, orally for group o and nasally for group n, with 3 × 10^8^ CFU of Y. enterocolitica Y09AL405. Following the same procedure, the six control piglets in group c were inoculated with 1:10 BHI-TS reference solution. The day of inoculation was noted as day 0. ◊, time point where surveillance was recorded and samples collected. At each time point of necropsy, samples from the tongue, tonsil, esophagus, mesenteric lymph nodes, spleen, liver, and intestinal contents of duodenum, jejunum, and ileum were collected.

The experimental setup is shown in Fig. 1. To study the dynamic of pig colonization, a pYV^+^ strain isolated from pig was used. Indeed, this genotype is often detected at farm level and is responsible of the majority of human yersiniosis. Our first goal was to determine the effect of the *Ye* inoculation method (oral [group o] versus nasal [group n]) on the growth and the health status of inoculated pigs. After inoculation, all pigs remained healthy throughout the trial irrespective of their challenge status, and they did not show any clinical signs suggestive of *Ye* infection, such as hunched posture, bristling, anorexia, or diarrhea. No lesions were observed postmortem. The mean weight of the pigs increased from 20.3 ± 2.5 kg 7 days before inoculation to 46.1 ± 4.9 kg at 21 days postinoculation (dpi) and to 60.8 ± 6.4 kg and 84.8 ± 9.0 kg at 35 dpi and 56 dpi, respectively. Weight did not differ significantly between the three groups (control group [group c], group o, and group n) during the study (*P > *0.05 at each dpi). Moreover, no decrease in feed consumption was observed in the inoculated groups after inoculation (data not shown). Before inoculation, mean body temperature (39.6 ± 0.2°C) did not differ significantly between the groups (c, o, and n) (*P > *0.05 at each dpi). After inoculation, the mean body temperature was similar (39.5 ± 0.3°C), and no pyrexia was observed in the control group. Nevertheless, the mean body temperature tended to be higher in the inoculated groups at 2 and 3 dpi (see Fig. S1 in the supplemental material) (*P < *0.05 at 3 dpi), but no statistically significant differences were observed following pairwise comparisons (*P > *0.05 at each dpi). Overall, these results indicate that after inoculation, all pigs remained healthy throughout the trial irrespective of their challenge status, as has been observed in cases of natural *Ye* infection on farms.

### *Ye* infection leads to a colonization pattern of three periods.

**FIG 2:**
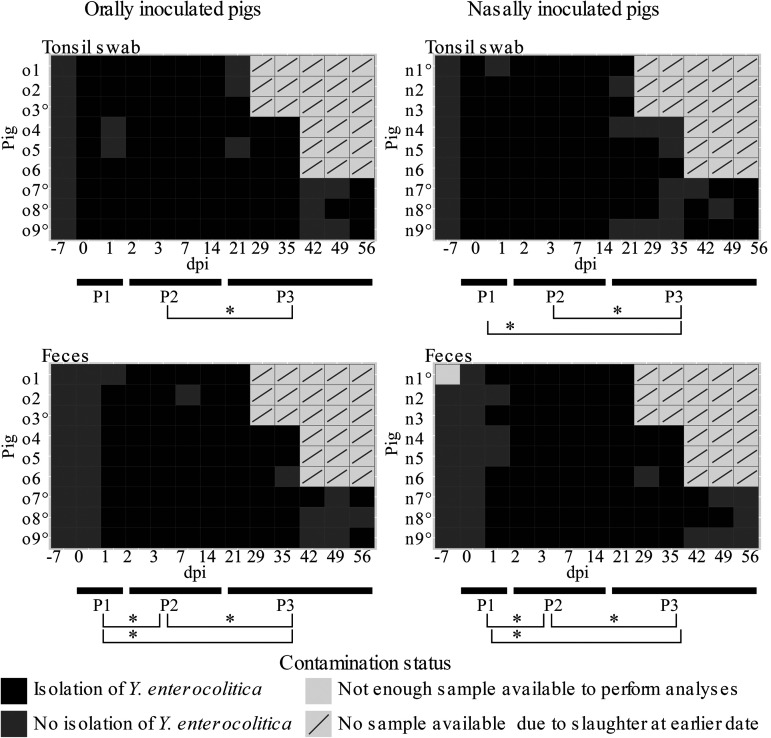
Contamination scheme and individual results of the isolation of Y. enterocolitica from feces and tonsil swabs over a 56-day period after oral (o) or nasal (n) inoculation of pigs with the bacteria. °, pig with samples for MLVA and the plasmid genetic stability study (Fig. 6 and 7). The three periods postinoculation (P1, P2 and P3) are indicated, and asterisks and brackets below the panels show when two periods are statistically significantly different (*P < *0.05, Fisher test).

Asymptomatic carriage of *Ye* has been reported at different pig ages, but the pattern of colonization just after infection has not yet been described. Because tonsils and feces are regarded as the samples of choice to monitor *Ye* status in pigs, the dynamic of *Ye* colonization was first assessed by microbiological analysis of feces and tonsil swabs throughout the trial (Fig. 2). No *Ye* was recovered from samples cultured before the 18 pigs were inoculated or during the trial from the samples collected from the 6 control pigs. Just after inoculation (0 dpi) and until 56 dpi, colonization of tonsil surfaces by *Ye* and *Ye* fecal excretion were determined concomitantly for the pigs, leading to a total of 336 analyzed samples. *Ye* was detected in 86% (145/168) and in 78% (131/168) of the tonsil swabs and feces samples, respectively.

**FIG 3:**
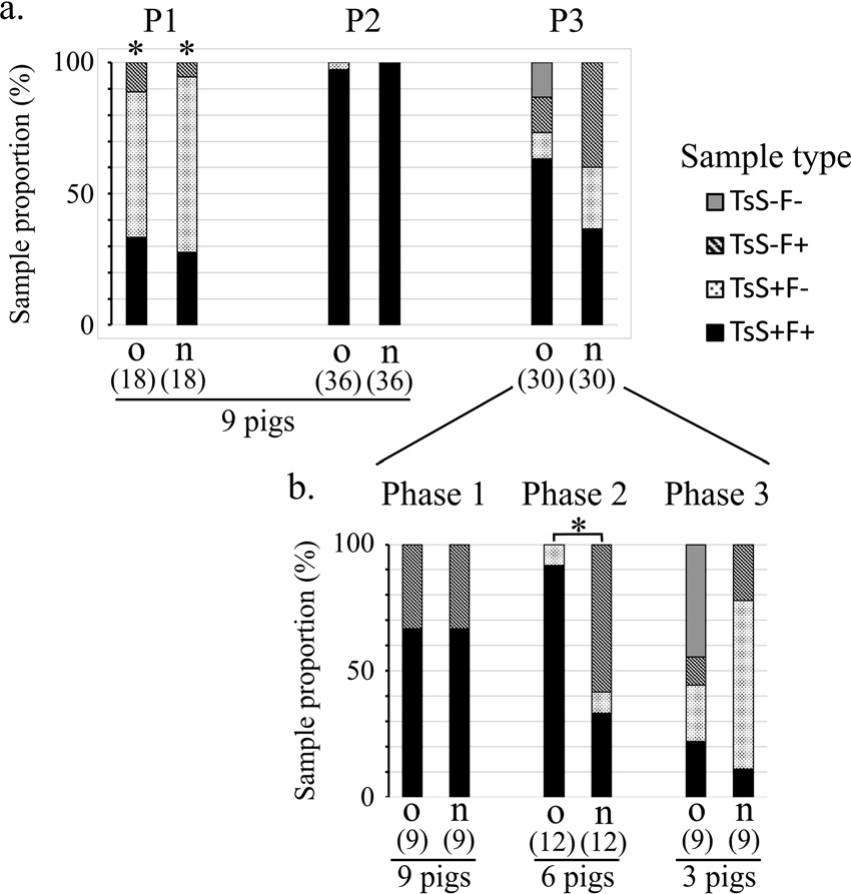
Results of the detection of Y. enterocolitica in tonsil swabs (TsS) and feces (F) samples during the three periods and the three phases of the P3 period in pigs inoculated orally (o) and nasally (n). (a) During the P1 (from 0 to 1 dpi), P2 (from 2 to 14 dpi), and P3 (from 21 to 56 dpi), 9 pigs were tested on each day. (b) During the P3 phases, phase 1 (21 dpi), phase 2 (from 29 to 35 dpi), and phase 3 (from 42 to 56 dpi), 9, 6, and 3 pigs, respectively, were tested. Total sample numbers are shown in parentheses. An asterisk indicates that for a given animal, *Ye* detection by tonsil swab samples (TsS) and feces (F) differed significantly (*P* < 0.05, McNemar’s chi-squared test). The square bracket with an asterisk indicates that results differed significantly between pigs inoculated orally and nasally (*P* < 0.05, Fisher test).

The time course of detection in tonsil swabs and in feces varied over time, depending on the nature of the sample. Three periods (P1, P2, and P3) were observed postinoculation (Fig. 2). The first period (P1, days 0 and 1 postinoculation [Fig. 1]) corresponded to the appearance of bacteria in tonsils or in feces. As early as 4 h after inoculation (0 dpi), the 18 orally and nasally inoculated pigs were positive for *Ye* in tonsils, irrespective of the inoculation route. Compared to tonsils, the first detection of *Ye* in feces appeared slightly later, between 24 h and 48 h after inoculation. At 1 dpi, most pigs (14/18) excreted *Ye* in their feces, and all of them at 2 dpi. A statistically significant difference (*P < *0.05) between feces and swabs was therefore observed during the first 24 h, before the establishment of *Ye* in tonsils and feces (Fig. 3a). The second period (P2), between 2 and 14 dpi, was characterized by the presence of *Ye* concomitantly in tonsils and in feces. During this period, *Ye* was detected in all tonsils (72/72) and in almost all feces samples (71/72), irrespective of the inoculation route (Fig. 2). In the third period, after 14 dpi (P3), for 6 pigs there was no detection and for the remaining 3 pigs, necropsied at 56 dpi, intermittent detection occurred (Fig. 2). Compared to P2, where almost all samples were positive for *Ye*, no *Ye* was detected in 33% (20/60) and 23% (14/60) of tonsil swabs and feces samples, respectively. During this period, *Ye* was detected in one, both, or none of the matrices tested (Fig. 2a). After 21 dpi, the dynamic of infection was followed for 6 pigs until 35 dpi and for three of them up to 56 dpi (Fig. 1). The pattern of detection varied from one pig to another during this period. *Ye* detection may cease as early as 21 dpi. Indeed, at 21 dpi and for each inoculation group, *Ye* was not detected in tonsil swabs for 3/9 pigs, although its presence was detected in all pig feces samples. Intermittent detection was observed for 3/6 pigs followed up to 35 dpi and for all the 6 pigs followed up to 56 dpi. Depending on the inoculated pig, periods with no detection of *Ye* lasted for 1 to 3 weeks. This concerned pigs from both groups (o and n) and both matrices (tonsil surfaces and feces). Thus, during P3, *Ye* was detected intermittently and sometimes alternatively in tonsil swab and feces samples.

### The presence of *Ye* tends to be differentially detected after nasal inoculation during P3.

Whatever the inoculation route, the type of sample and the proportion of each type were similar in P1 and P2 but seemed different in P3 (Fig. 3a). In contrast to P1 and P2, where 9 pigs were studied through each period, the number of pigs decreased during P3 because necropsies were done. To further describe the P3 period by taking into account the variation in pig numbers, the period was divided into 3 phases (phase 1, phase 2, and phase 3) corresponding to the study of 9, 6, and 3 pigs in each group, at 21 dpi, 29 to 35 dpi, and 42 to 56 dpi, respectively (Fig. 2 and 3b). At the beginning of P3 (phase1, 21 dpi) and similarly in both groups o and n, *Ye* was detected in feces (F) but not in tonsil swabs (TsS) of 3 pigs (TsS^−^ F^+^) and in both matrices of 6 pigs (TsS^+^ F^+^) (Fig. 2 and 3b). During phase 2, the colonization tended to differ with statistical significance (Fig. 3b) (*P = *0.002) according to the route of inoculation. Indeed, *Ye* was concomitantly found in feces and tonsils, with a higher frequency of detection for the 6 orally inoculated pigs (91.7%) than the 6 nasally inoculated pigs (33.3%). The detection profile (TsS^−^ F^+^) was observed only for nasally inoculated pigs (5/6 pigs) (Fig. 2 and 3b) during phase 2. During the end of P3 (phase 3), the absence of detection in feces and tonsil swabs was observed at one or two time points of sampling for the 3 orally inoculated pigs and for none of the 3 nasally inoculated pigs. These results allowed us to state the hypothesis that the colonization of pigs by *Ye* could vary in P3 depending on the route of inoculation. Nevertheless, more data are required to confirm this hypothesis.

### *Ye* colonization occurs mainly in the oral cavity and tends to decrease with time depending on the samples.

**FIG 4:**
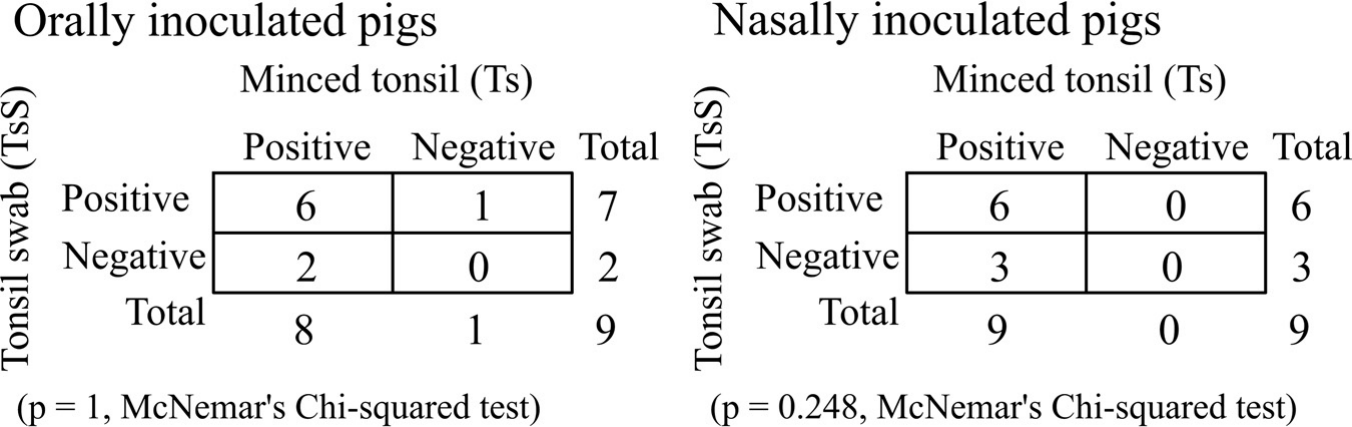
Agreement of the detection of Y. enterocolitica in tonsil samples by swabbing and by mincing at 21, 35, and 56 dpi in pigs inoculated orally and nasally.

At 21 dpi, the mean weight of pigs was 46.1 ± 4.9 kg, which corresponds to the weight of pigs at the beginning of the fattening period. In animal husbandry, the presence of *Ye* in tonsils and feces is detected mainly during the fattening period, but few data on the colonization status of internal organs before slaughtering have been collected. To further characterize the *Ye* colonization process, 6 internal organs and 3 intestinal compartments were sampled in 3 pigs at 21, 35, and 56 dpi (Table 1). No organs or intestinal contents from control pigs were positive for *Ye.* In inoculated pigs, irrespective of the inoculation route, no spleen or liver samples were found to be positive for *Ye* during the trial. In contrast, *Ye* was recovered from the tongue (T), tonsils (Ts), esophagus (E), and mesenteric lymph nodes (M) and from the different intestinal compartments, namely, duodenum (D), jejunum (J), and ileum (I). Whatever the inoculation route was, the presence of *Ye* in tonsils (organ samples) was detected at each time point (21, 35, and 56 dpi) and for all inoculated pigs (Table 1). Moreover, for the 5 pigs that were negative after the tonsils were swabbed at 21 and 35 dpi, minced tonsils were positive (Fig. 1). The statistical analysis performed on the 18 tonsils analyzed by both methods (swabbing and mincing) indicated no significant differences between swabbing and mincing tonsils to detect the presence of *Ye* (*P > *0.05) (Fig. 4). In conclusion, regardless of the inoculation route, the presence of *Ye* was maintained in the oral cavity, and the frequency of detection was maximal at 21 dpi and tended to decrease subsequently.

**TABLE 1:** Numbers of samples positive for Y. enterocolitica at necropsy at 21, 35, and 56 dpi and in total after oral or nasal inoculation of pigs^*a*^

Group	Pig	Necropsy date (dpi)	No. of positive samples (total)							
			Ts	T	E	D	J	I	M	Total
Oral	o1, o2, o3°	21	3	3	3	3	2	2	2	18 (21)
	o4, o5, o6	35	3	2	0	0	0	0	0	5 (21)
	o7°, o8°, o9°	56	3	1	0	0	1	0	0	5 (21)
	Total		9 (9)	6 (9)	3 (9)	3 (9)	3 (9)	2 (9)	2 (9)	28 (63)
Nasal	n1°, n2, n3	21	3	3	1	0	2	1	0	10 (21)
	n4, n5, n6	35	3	1	1	0	0	0	0	5 (21)
	n7°, n8°, n9°	56	3	1	0	0	0	0	0	4 (21)
	Total		9 (9)	5 (9)	2 (9)	0 (9)	2 (9)	1 (9)	0 (9)	19 (63)

a Ts, tonsil; T, tongue; E, esophagus; D, contents of duodenum; J, contents of jejunum; I, contents of ileum; M, mesenteric lymph nodes. Pig IDs indicate pigs with samples for the study of *Ye* genetic stability (Fig. 6 and 7). No organs or intestinal contents from control pigs were positive for Y. enterocolitica.

### Antibody response occurred between 7 and 14 dpi and up to 56 dpi.

**FIG 5:**
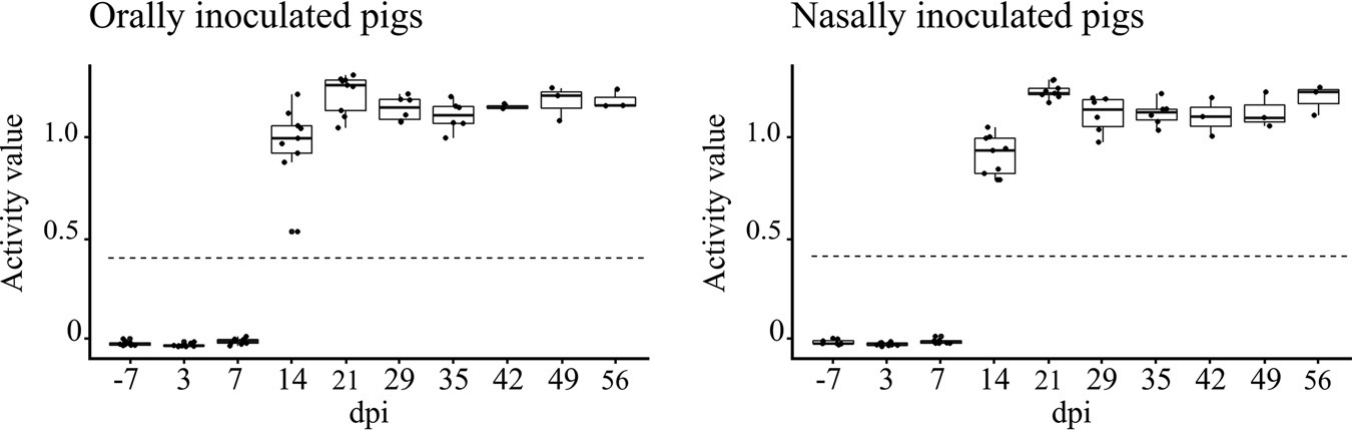
Activity value of ELISA serum IgG antibodies to Y. enterocolitica in the orally and nasally inoculated pigs. The activity value was calculated based on *A*, as follows: (*A*sample − *A*neg)/(*A*pos − *A*neg). The cutoff value is indicated by the dashed line.

Serological responses of pigs against *Ye* were monitored by enzyme-linked immunosorbent assay (ELISA) throughout the trial. None of the pigs in the control group seroconverted. The mean serological titers of piglets orally and nasally inoculated with *Ye* were similar (Fig. 5). No IgG antibodies were detected until 7 dpi. Seroconversion occurred between 7 and 14 dpi, at which time point 100% of the piglets were seropositive. A strong increase in the activity value (ae) was observed between 14 dpi (ae_mean_ = 0.91 ± 0.17) and 21 dpi (ae_mean_ = 1.20 ± 0.08), and antibody levels remained high (ae ≥ 1.0) throughout the trial for all tested pigs, irrespective of their bacteriological status.

### Influence of pig colonization on the results of genotyping and emergence of genetic modifications.

PFGE and MLVA are used to characterize strains and group them into clusters. Strains sharing a PFGE pattern or an MLVA type are considered identical. The genetic stability of the strain during colonization was thus assessed by PFGE and MLVA methods. The absence of *Ye* in control pigs confirmed that the different *Ye* isolates recovered from the orally and nasally inoculated pigs derived only from the inoculated strain.

The genetic pattern of the strains was determined by the NotI PFGE profile for 23 isolates collected from *Ye-*positive orally inoculated pigs (Table S2). The genetic pattern obtained was identical to that of the inoculated strain, regardless of the sample type and pig tested (Fig. S2). During the 56 days of the trial, no genetic variation of the *Ye* strain was observed by PFGE analysis.

**FIG 6:**
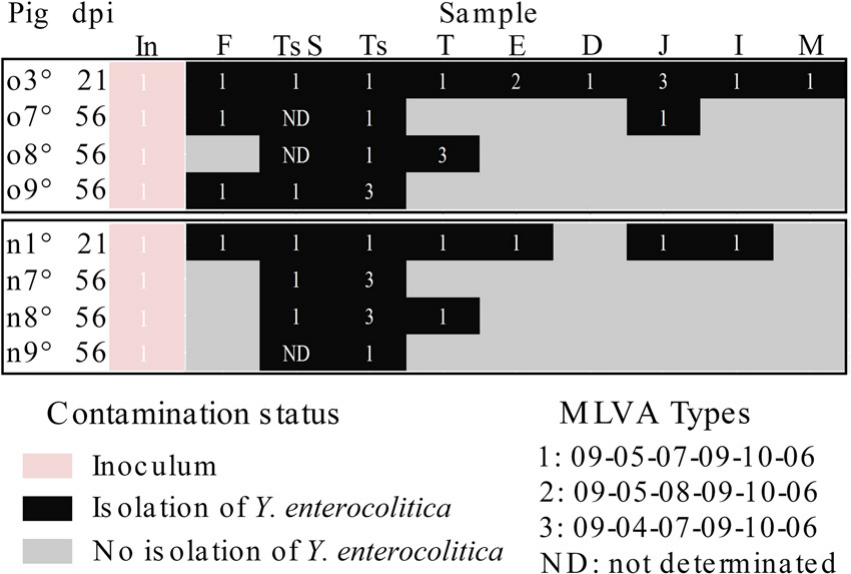
Genetic stability of Y. enterocolitica from samples analyzed at 21 or 56 days after oral (o) or nasal (n) inoculation of pigs, as determined by MLVA typing. MLVA type was determined for the inoculum (In) and isolates from samples collected from pigs necropsied at 21 and 56 dpi. Samples originated from feces (F), tonsil swabs (TsS), tonsils (Ts), tongues (T), esophagi (E), contents of the duodenum (D), jejunum (J), and ileum (I), and mesenteric lymph node (M).

Contrary to PFGE, MLVA of the 30 isolates collected from pigs at 21 or 56 days after oral or nasal inoculation showed three MLVA types instead of one as expected (Fig. 6). MLVA type 1 (09-05-07-09-10-06), corresponding to the type of the inoculated strain, was recovered from 80% (24/30) of all tested isolates in all pigs, organs, and intestinal content samples. The other two MLVA types detected, type 2 (09-05-08-09-10-06) and type 3 (09-04-07-09-10-06), were recovered from 3% (1/30) and 17% (5/30) of the tested isolates, respectively. These two new MLVA types varied from the original MLVA type by only one locus, locus V5 for MLVA type 2 and locus V4 for MLVA type 3. These results indicate that isolates of up to three MLVA type can be found in one pig. These new MLVA types appeared regardless of the inoculation route and were recovered from different organs: four were recovered from the oral cavity (tonsil and tongue), one from the esophagus, and one from jejunum content. Importantly, *Ye* isolates recovered from tonsil and tongue exhibited MLVA types 1 and 3, respectively (Fig. 6); however, they shared the same PFGE profile. This result emphasizes the importance of using MLVA typing because it has higher discriminatory power than PFGE. In summary, this is the first time genetic modification of *Ye* during colonization has been demonstrated.

### Stability of virulence traits during pig colonization.

The inoculated strain carried the chromosomic gene *ail* and the plasmid gene *yadA*, two genes associated with virulence traits. Even though no genetic variations concerning the *ail* PCR product (A^+^) were detected, isolates without the *yadA* virulence PCR product (Y^−^) were detected, as early as 21 dpi. This concerned exclusively the three pigs inoculated by the oral route and the samples corresponding to feces and duodenal and jejunum contents. The three pigs concomitantly harbored isolates with conserved inoculum virulence traits (A^+^ Y^+^) and isolates that had lost the original *yadA* marker (A^+^ Y^−^).

**FIG 7:**
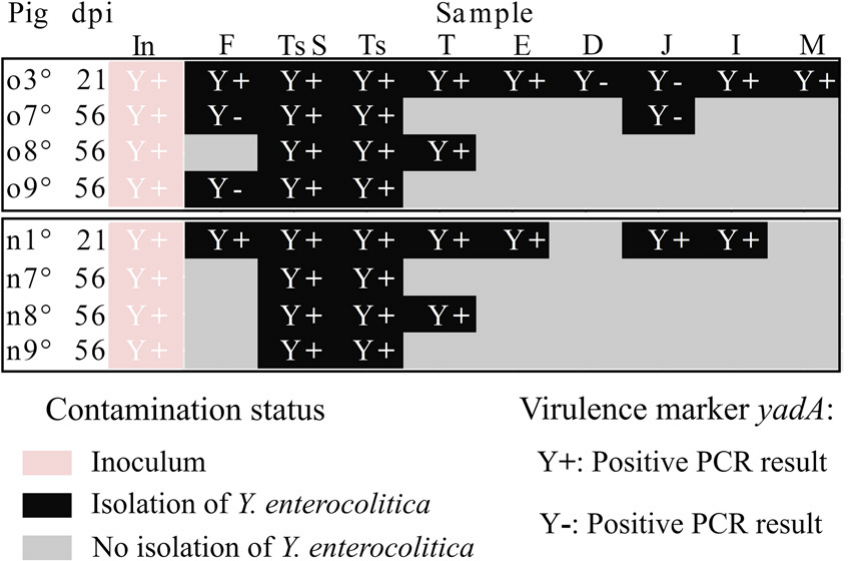
Genetic stability of Y. enterocolitica from samples analyzed at 21 or 56 days after oral (o) or nasal (n) inoculation of pigs, showing presence (Y+) or absence (Y-) of the *yadA* (plasmid) virulence PCR marker. The presence of *yadA* was determined for the inoculum (In) and isolates from samples collected in pigs necropsied at 21 and 56 dpi. Samples originated from feces (F), tonsil swabs (TsS), tonsils (Ts), tongues (T), esophagi (E), contents of the duodenum (D), jejunum (J), and ileum (I), and mesenteric lymph nodes (M).

Comparison of the genotyping and PCR results (Fig. 6 and 7) indicated that the inoculum virulence traits (A^+^ Y^+^) were detected in MLVA type 2 or 3 isolates, and indicated the loss of the *yadA* marker (A^+^ Y^−^) in MLVA type 1 or 3. These results demonstrate that the presence or absence of the *yadA* plasmid marker was independent of the MLVA type of the isolate.

## DISCUSSION

To understand and prevent transmission of human-virulent *Ye* on pig farms and the routes of contamination, the choice of an experimental model of infection is crucial. We chose Large White pigs, one of the main livestock species used in crossbreeding in the pig industry (23), and a *Ye* 4/O:3 strain harboring the *ail* gene and the virulence plasmid pYV, as it is the most commonly reported strain from human cases and is detected at the pig level (2, 12). At the farm level, contamination may occur at different stages of breeding. Although pigs appear to be contaminated mainly during the fattening period (9, 15), infection may also occur and spread among piglets (24). In order to examine colonization by *Ye* during the life of a pig, we developed a model in which 8-week-old piglets were inoculated. Like on most farms, pigs were reared in pens with slatted floors (25). The experimental conditions applied in our study were controlled and resulted in growth similar to that observed on farms, as no contamination, abnormal behavior, or physiological defects were observed in pigs in the control group during the trial.

Depending on the experimental procedure, clinical responses to inoculation with the *Ye* 4/O:3 pYv^+^ strain may range from absence of symptoms or mild illness to death (26, 27). An increase in pig body temperature demonstrated the development of an inflammatory process during the first week following oral challenge with 5 × 10^10^ CFU of 4/O:3 pYv^+^ *Ye* (27). In our study, the increasing temperature at 2 and 3 dpi may be the result of an early inflammatory reaction that quickly ends. The severity of host response to a challenge may be dose related (26) and may also depend on *Ye* strain properties (28). In our protocol, piglets were inoculated with a hundred-times-lower number of bacteria than the protocol used by Najdenski et al. (27) and with a strain originating from an asymptomatic pig. Despite the human virulence features harbored by the strain used in this study, no clinical signs were expected. Our results were consistent with asymptomatic infection as described for infected pigs under natural breeding farm conditions (13, 15).

Pigs are burrowing animals. Naturally, acquired *Ye* infection may typically occur following inhalation or ingestion of the bacteria via the snout or the mouth. Although previous studies have identified snout contacts as a possible risk factor for pigs to become contaminated with *Ye* (29), no experiments using the nasal inoculation route have been reported so far. Here, we report persistent contamination regardless of the inoculation route, e.g., oral or nasal, demonstrating that *Ye* pig contamination may occur via the introduction of the bacteria not only via the mouth but also via the nares.

It is essential to understand the dynamics of pig colonization to preclude contamination and to prevent its expansion. Considering colonization in the digestive system and in extraintestinal sites, concomitantly with the immune response, this longitudinal experimental infection highlighted three periods in the colonization process. The first period corresponded to the 24 h following ingestion or inhalation of *Ye* and included the first establishment steps of *Ye* colonization in pigs. Detection of *Ye* in tonsils 4 h after inoculation, in line with observations made by Thibodeau et al., and the absence of *Ye* recovery from feces before 24 h postinfection support the establishment of an early colonization process in pigs, especially in the tonsils (30). Therefore, tonsils appear to be more appropriate than feces to detect recent *Ye* contamination. During the second period of colonization, which lasted from 2 dpi to 14 dpi, pigs harbored *Ye* in their tonsils and concomitantly excreted *Ye* in their feces. Previous studies reported that almost all asymptomatic pigs contaminated with a *Ye* 4/O:3 p^+^ strain excreted *Ye* in feces (28, 31, 32) from 5 to 21 dpi, with a high concentration of the strain (2 to 6 log CFU/g) in feces (31, 32) and the presence of *Ye* in different intestinal and extraintestinal organs (28). These results suggest that this period corresponds to the expansion of *Ye* in the pig body, allowing generalized colonization of the digestive system and extraintestinal organs, such as tonsils and mesenteric lymph nodes, without generating clinical signs. The persistence of *Ye* in tonsils and feces indicates that pigs could be an important source of contamination during this period. During the third period, which occurred after 21 dpi, a decrease in colonization was observed in the digestive system and internal organs with intermittent *Ye* detection in pigs. Other studies have reported similar results. It was observed that most bacteria were cleared from intestinal and extraintestinal sites 6 h postinoculation and that organ colonization was lower at 21 dpi than at 3 and 14 dpi (28, 30). However, the transient detection in digestive tract and internal organs could correspond to a presence/absence of *Ye* in organs or a variation of the number of *Ye* organisms (low versus high number). It is indeed possible to quantify *Ye* in highly contaminated samples using direct plating (28, 33). However, in samples with low levels of contamination, an enrichment step is needed, especially in the presence of significant background flora, such as in feces or intestinal contents. To favor the detection of *Ye* in the present study, enrichment in Irgasan-ticarcillin-potassium chlorate (ITC) medium was carried out. This medium promotes *Ye* growth and is recommended by the international standard ISO 10273:2017 (21). The sample size inoculated in the ITC medium was 10 times (feces) to 25 times (organs and intestinal contents) higher than the recommended one in the standard ISO 10273:2017 and similar to the one reported in previous studies (21, 30, 33). Observations during the three periods support the idea that regardless of the inoculation route, colonization of the digestive tract and internal organs changed over time, while it was persistent in the oral cavity and tonsils. Further studies are needed to develop a quantitative approach to specifying the level of sample contamination during the third period.

In our study, the presence of *Ye* was not always detected by tonsil swabbing, but it was confirmed by assessing minced tonsils. Nevertheless, no statistically significant differences were observed between the results obtained by swabbing and by mincing tonsils. Higher isolation rates have generally been obtained when the tonsils from fattening pigs were homogenized rather than swabbed, meaning that the number of *Ye* decreased on tonsil surfaces and that *Ye* may persist better in deep tonsil tissue (33, 34). Changes in tonsil colonization may then occur during the third period. This hypothesis could be supported by the fact that microscopy and microbiota analysis have indicated that *Ye* is able to colonize deep tonsil tissue and form microcolonies (35–37). Serological evidence also supports this hypothesis, because a close association between seropositivity and isolation of *Ye* from minced tonsils has been shown (38). After 21 dpi, *Ye* was not detected in all swab samples, but systematically high serological values obtained at each sampling time point may suggest the presence of *Ye* in tonsils, possibly in deeper tonsil tissue. In our study, the absence of *Ye* detection in tonsils may therefore be due to the method used. During P3, the intermittent detection of *Ye* in tonsil swabs or in feces samples may indicate either stable but low numbers of *Ye* close to the detection limit of the method or an intermittent absence of *Ye* on tonsil surfaces and in feces. Indeed, feces, internal organs and tonsil surfaces are often less contaminated with *Ye* after 21 dpi; therefore, the probability that these pigs contaminated other carcasses during the slaughter process is low. Although 3 periods were observed regardless of the inoculation route, the latter may influence the colonization ability of *Ye*. Actually, the detection of *Ye* in tonsil swabs and feces tended to be different during phase 2 and 3 of P3. Because of the low number of pigs involved in the trial, further studies are needed to confirm the variability in the dynamic of colonization induced by the route of inoculation. In the present work, seroconversion occurred between 7 and 14 dpi and lasted until 56 dpi (end of the experiment). These results are consistent with those found in several experimental and in-field studies (15, 28, 32). Seroconversion has also been detected later, e.g., at up to 7 weeks in naturally infected pigs and between 3 and 8 weeks in artificially contaminated pigs. In artificial contamination, variability could be related to the efficiency of colonization (14, 39). In our trial, the serological values remained high until the end of the trial. In another study, a persistent serological reaction was detected for up to 70 days after infection (32).

These results strengthen the idea that serology could be a useful tool to identify either an early or a late stage of *Ye* infection.

In *in vivo* studies, the genetic stability of strains has been shown to vary depending on the strain and the species (40–42). In our study, the impact of colonization on the chromosomal and plasmid content of isolates was screened using PFGE, MLVA, and the virulence markers *ail* and *yadA*.

NotI PFGE has been used to discriminate BT4 isolates originating from pigs (43–45). Because two different pulsotypes may be found in one pig (46), the question of the emergence of new clones due to mutation could be raised. However, the presence of NotI-dominant pulsotypes observed over months to several years in pigs (43, 45) may also indicate stable transmission involving the same strain, without genetic changes. In the present longitudinal study, no variations in genetic content were detected either at the chromosomal level with PFGE or at the gene level using the *ail* marker. The systematic detection of *ail* in this study confirmed the stability of this marker, which is consistent with studies investigating its naturally high prevalence in pigs (85% to 100%) and its significant role in the process of host colonization (6, 9, 13, 19, 43, 45, 47). Although *ail* profiling and PFGE typing indicated that the inoculated strain was transferred without large DNA rearrangement or genetic variation during colonization. *ail* profiling and PFGE typing results do not completely preclude the possibility that some genetic mutations may occur in the *Ye* genome during colonization. Most isolates harbored the same genetic content as the inoculated strain, but 33% of them (10/30) showed genetic variations based on MLVA typing and plasmid profiling.

Our study generated epidemiologically relevant data on the genetic variation to be considered when using the *Ye* MLVA scheme. The ability of MLVA to distinguish among isolates that appeared to be closely related by PFGE has already been reported (10, 12, 44). However, previous data obtained using PFGE or MLVA have shown that *Ye* is stable genetically. The same *Ye* PFGE pattern and MLVA type have been recovered at different times points from infected humans or from pig farms (44, 48, 49). Virtanen et al. observed no changes in the MLVA types of 4/O:3 in pig feces, up to 91 days after pig contamination (24). Here, we showed that VNTRs may evolve and lead to the emergence of new MLVA types during pig colonization that was initially caused by a single clone. The present work is thus the first to demonstrate that genetic modification may occur in *Ye* strains during host colonization. Because MLVA is used as an epidemiological tool to investigate *Yersinia* outbreaks and *Yersinia* in pigs (49–51), this aspect has to be taken into account to establish isolates’ relatedness for MLVA data interpretation.

The MLVA scheme for *Ye* is composed of 6 loci having different discriminatory powers (10, 12, 44). In the present study, MLVA type variation was observed only in 2 loci, V4 and V5. These findings suggest that the *Ye* MLVA scheme contained four loci with a relatively high genetic stability and two with a high genetic dynamic. From a molecular point of view, the ability of these markers to detect differences increases the likelihood of genetic polymorphisms and provides more subtle diversification to discriminate between very closely related isolates.

The variations in V4 and V5 correspond to a deletion or to an addition of one repeat, respectively. Thus, variant isolates differed from the inoculated strain by a single repeat, suggesting that variations that occur during pig colonization are likely to involve a small number of repeats. Interestingly, the predicted functions of the translated VNTR sequences for V4 (MLVA type 3) and V5 (MLVA type 2) are entirely located within coding regions of putative genes (48) (https://blast.ncbi.nlm.nih.gov/Blast.cgi). For MLVA type 3, the change is predicted to produce the modification of the N-terminal sequence of a glycerate kinase protein which plays a key role in carbohydrate metabolism (52). Mutations in MLVA type 2 isolates are predicted to generate an increase in the variable-length peptide repeats of the periplasmic SanA protein, which has been implicated in functions such as vancomycin resistance, envelope integrity, and anaerobic respiration (53–55). Therefore, these variabilities may have a potential role in modulating gene functions and thus may contribute to modification of *Ye* adaptation and persistence in pigs.

Over time, variants appeared, and it will be interesting to test whether they show selective advantages. Interestingly, the two MLVA variant isolates have different profiles of detection. MLVA type 3 isolates were detected in 5 pigs, mainly in the oral cavity (tongue and tonsils) and up to 56 dpi, while MLVA type 2 was detected in only one pig at 21 dpi, in a transient organ, the esophagus. Remarkably, mutation in V4 emerged independently at least twice, as MLVA type 3 isolates were detected in pigs located in different confined animal houses. It could be speculated that MLVA type 3 isolates may have some selective advantages compared to MLVA type 2 isolates. Deciphering the ability of these variants to colonize pigs may increase knowledge in mechanisms of host-pathogen interaction and adaptation.

It is noteworthy that MLVA variants were detected in pigs concomitantly with the MLVA type corresponding to the inoculated strain (MLVA type 1). These variants were detected as early as 21 dpi and up to 56 dpi. MLVA type 1 was detected in all sites tested. The detection of MLVA variants in 4 different sites (tonsils, tongue, esophagus, and jejunum) may be due to spontaneous and independent appearance of variants in different sites or the migration of *Ye* MLVA variants from one site to another. The MLVA variants were detected mainly in the oral cavity (tongue and tonsils) but not in feces. The oral cavity may favor the appearance and expansion of new MLVA variants, since this site was persistently contaminated with *Ye* strains. Nevertheless, in pigs reared in groups, we cannot determine whether all the mutations arose independently *in vivo* in one pig or if some of the mutant isolates passed between pigs and were acquired through a contamination process. Although no trend associating the genetic variation and the sampling origin and period was found, the oral cavity could be the site where new MLVA variants could emerge and/or could easily be detected.

Variants were recovered from 5 pigs among the 12 studied. Profiles differ from one pig to another. We showed that pigs were able to carry up to three MLVA types at different sites. Intrinsic individual variability may influence the emergence of new MLVA variants. It would be interesting to investigate these results further to elucidate the ability of *Ye* to adapt and colonize the host.

Genetic instability was also observed by screening the plasmid *yadA* marker. Among the 12 pigs, three concomitantly harbored isolates with and without the original *yadA* marker, depending on the sampling site. The absence of *yadA* likely occurred during colonization rather than during laboratory handling, as reported previously (56). Moreover, to overcome possible laboratory artifacts, we carried out manipulations at temperatures under 37°C and avoided multiple streaking. Interestingly, *yadA* isolates have been detected in particular in the proximal part of the intestine, where bacteria harboring plasmids were present in lower concentrations (26).

No correlation was observed between the presence or absence of the *yadA* plasmid marker and the MLVA type of the *Ye* isolates, suggesting that the genetic modifications resulted from different processes of adaptation and indicating some genomic plasticity in *Ye* BT4 strains.

As a result, although several variants may be detected at the same time in a pig, they all resulted from slight genetic variations. Their concomitant presence with the inoculated isolates highlighted a moderate process of genetic evolution of the BT4 strain during pig colonization.

This study provides additional data generating knowledge on pig colonization by 4/O:3 *Ye*, and we propose an experimental model of infection. Pig contamination occurred orally or nasally and led to asymptomatic but generalized colonization. Seroconversion appeared to predate the regression of the *Ye* colonization area. Neither microbiological detection by tonsil swab or feces nor serological data precisely predict the pig status when used independently. By combining the data, three periods corresponding to three contamination statuses of the pig were identified. This important outcome could make it possible to determine potential interventions for preventing *Ye* contamination from farms to slaughterhouses. The virulence properties of the bacteria were well conserved during the three colonization periods, but new clones may emerge and be concomitantly present in one pig.

The proposed controlled experimental infection model provides a new way to study pathogen-host relationships, such as *Ye* in pigs. More studies are needed to further investigate the steps involved in the persistence of contamination, to identify new markers for accurate definition of pig contamination status, and to predict the possible genetic variations of *Ye* and their impact on pig colonization. Using the proposed experimental *Ye* infection model in pigs in combination with whole-genome sequencing (WGS) methods would make it possible to address these topics.

## MATERIALS AND METHODS

### Ethics statement.

All animal work was performed in strict accordance with directive 2010/63/EU and validated by the Ethics Committee in Animal Research (Anses/ENVA/UPEC) no. 16 of the French Ministry of National Education, Higher Education and Research (license APAFIS 2697-2015110409457994). The trial protocols were approved under reference 12-028.

### Animals and housing.

The trial was conducted in 6-week-old specific-pathogen-free (SPF) Large White piglets naturally born from sows controlled for the absence of major swine viral and bacterial pathogens, including *Yersinia* spp. (see Table S1 in the supplemental material for the full list of tested pathogens).

The experiment was carried out in biosafety level 2 (BSL2) animal facilities maintained under negative pressure at ANSES, Ploufragan-Plouzané-Niort laboratory, France. Three confined animal houses were used, each initially housing 6 or 9 piglets. Each animal house had a separate ventilation system and was operated under an all-in all-out system. Strict biosecurity measures were implemented to avoid contamination of the pigs, including the use of an air filtration system and airlocks for each unit, unit-specific clothes, and compulsory showering before and after visiting the pigs.

The trial includes 24 piglets born from 3 sows. To avoid a sampling effect, piglets were stratified by gender, weight, and the sow they originated from and were assigned to group o (9 piglets), n (9 piglets), or c (6 piglets) and located in 3 separate animal houses (Fig. 1). During the trial, the pigs were fed normal rations *ad libitum*.

### Inoculated *Ye* strain.

Strain Y09AL405, used for the experimental model, was isolated in 2009 from naturally contaminated pigs at a slaughterhouse (57). This is a BT4/O:3 strain that harbors the pYV plasmid and the chromosomic virulence genes *ail*, *inv*, *myfA*, and *ystA*. Prior to inoculation, an overnight culture of Y09AL405 was diluted 100-fold in brain heart infusion broth (BHI) and incubated for 2 h at 30°C with agitation (250 rpm). The fresh culture was then diluted 10-fold in tryptone salt solution (TS), to obtain a solution containing 3 × 10^7^ CFU/mL of *Ye*.

### Experimental design.

The piglets were allowed a 7-day socialization period, and their *Ye-*negative status was confirmed at 7 weeks of age by bacteriological and serological examination. Eighteen piglets were then inoculated at 8 weeks of age, orally for group o and nasally for group n, with 10 mL/piglet (total inoculated dose, 3 × 10^8^ CFU of *Ye*) (Fig. 1). Following the same procedure, the six control piglets in group c were inoculated with 10 mL of 1:10 BHI-TS reference solution. The day of inoculation was noted as day 0.

Pigs were tested for *Ye* contamination status by collecting individual tonsil swabs (TsS) and fecal samples (F), 7 days before inoculation and at 0, 1, 2, 3, 7, 14, 21, 29, 35, 42, 49, and 56 days postinoculation (dpi). The samples recovered at 0 dpi correspond to the time point of first feces excretion after inoculation, and this occurred less than 4 h after inoculation. Blood samples were taken from all pigs once a week from day 0.

Pigs were submitted to postmortem examination at 21, 35, or 56 dpi. At each time point, three pigs from groups o and n each and two pigs from group c were euthanized (anesthesia with 15 mg/kg of Zoletil [tiletamine and zolazepam; Virbac, Carros, France]), followed by bleeding.

Samples of the tongue (T), tonsil (TS), esophagus (E), mesenteric lymph nodes (M), spleen (S), liver (L), and contents of the duodenum (D), jejunum (J), and ileum (I) were collected for bacteriological analysis.

### Clinical surveillance.

Pigs were examined clinically on a daily basis (Fig. 1). This included external physical examination, behavior observation, and examination of stool consistency. The weight and rectal temperature of each animal were recorded once a week, starting from −7 until 56 dpi. Rectal temperatures over 40.0°C were considered hyperthermia. Feed consumption was recorded daily by measuring the leftover feed for each animal house, considering the animal house as the experimental unit.

### Isolation and identification of pathogenic *Ye*.

Samples were collected and stored for 24 h at 4°C before analysis. The presence of *Ye* was assessed for each sample according to a modified protocol from the standard ISO 10273:2017 and from the work of Fondrevez et al. (21, 57). Briefly, fecal samples were diluted 1:10 in peptone salt broth (bioMérieux, France); then, 1 mL was transferred to a tube containing 9 mL Irgasan-ticarcillin-potassium chlorate (ITC) broth (Bio-Rad, Marnes-La-Coquette, France). Minced organ samples and intestinal contents (2 g) or the brush for tonsil swabs was placed directly in 9 mL of ITC. The ITC enrichment broth was incubated for 48 h at 25°C. Streaking was done on cefsulodin-Irgasan-novobiocin (CIN) agar plates (*Yersinia* selective agar base and *Yersinia* selective supplement; Oxoid, Basingstoke, UK). After 24 h at 30°C, the plates were checked for the presence of typical colonies on CIN plates (red “bull’s-eye” colonies). A maximum of 4 characteristic colonies per sample were then streaked on Yersinia enterocolitica chromogenic medium (YeCM) (58). Colonies identified as possibly pathogenic (not blue on YeCM) were subcultured on plate count agar (PCA) (bioMérieux, France) and incubated at 30°C for 24 h. The isolates were stored in peptone glycerol broth at −80°C until further characterization.

The presence of the *ail* gene was screened for by PCR on isolates from the positive samples collected at 56 dpi on 3 orally and 3 nasally inoculated pigs (pigs o7°, o8°, o9°, n7°, n8°, and n9°, respectively, where “°” indicates pigs with samples for MLVA and the plasmid genetic stability study). The presence of the *yadA* gene was screened for by PCR on isolates from the positive samples collected at 56 dpi and at 21 dpi. One isolate per sample was tested, except when the PCR result was negative for the presence of the marker, in which case a second isolate was tested. As a result of this process, the absence of the *yadA* marker (*yadA* PCR product) was confirmed only after obtaining two negative PCR results, on two isolates. The PCR was done using a 0.3 μM concentration of the *ail* primers (59) and a 0.4 μM concentration of the *yadA* primers (60) in a final volume of 25 μL with SYBR green Jumpstart *Taq* ReadyMix (Sigma-Aldrich, St. Louis, MO, USA) at 1×. The cycling conditions were one cycle of 95°C for 3 min and 35 cycles of 94°C for 60 s, 58°C for 60 s, and 72°C for 60 s in a CFX96 real-time PCR detection system (Bio-Rad, Hercules, CA, USA). The melting point analysis was conducted by acquiring fluorescence data at the temperature ramp of 65°C to 95°C at 0.1°C intervals for 5 s.

### Serology.

Sera separated from blood were stored at −20°C until testing. Serum samples were screened for the presence of IgG antibodies against *Yersinia* outer membrane proteins (YOP) using a commercially available enzyme-linked immunosorbent assay (ELISA) kit (Pigtype Yersinia Ab; Qiagen, Leipzig, Germany), according to the manufacturer’s instructions with overnight incubation, and measuring absorbance (*A*) at 450 nm. The activity value (ae) was calculated based on *A* values, relative to the mean *A* value of the positive control (*A*pos) and negative control (*A*neg), as follows: ae = (*A*sample − *A*neg)/(*A*pos − *A*neg). Samples with activity values of 0.4 or higher were considered positive.

### Genetic stability of the inoculated *Ye* strain.

**(i) PFGE.** To study the genetic stability of the strain during the trial, PFGE was performed on the inoculated strain (used as the reference strain) and on 23 isolates collected from *Ye*-positive orally inoculated pigs at 0, 21, 35, or 56 dpi. One isolate per *Ye*-positive sample type was selected (Table S2).

PFGE analysis was conducted according to a previously described protocol (12). Salmonella enterica serovar Braenderup strain H9812 was used as a size marker to allow comparison of the PFGE profiles from different gels. Briefly, bacterial strains were subcultured on PCA at 30°C for 24 h. The culture was suspended in Tris-EDTA (TE) buffer (0.01 M TE buffer, pH 8.0) and adjusted to an optical density (600 nm) of 1.5. This suspension was then mixed with 1% agarose to prepare the plugs, which were incubated for 48 h at 50°C in a lysis solution (0.5 M Na_2_EDTA [pH 9], 1% *N*-lauryl-sarcosyl, 1 mg/mL proteinase K), and washed five times with TE buffer. DNA from Salmonella Braenderup or *Ye* was then digested with 40 U of XbaI or NotI restriction enzyme (Roche, Boulogne-Billancourt, France), respectively, for 6 h at 37°C. The electrophoresis lasted 27 h at 6.6 V with an initial switch time of 1.5 s and a final switch time of 18.0 s. Electrophoretic patterns were compared, and similarities between profiles were determined using BioNumerics (version 7.6; Applied Maths, Sint-Martens-Latem, Belgium) by constructing a dendrogram using the unweighted pair group method with arithmetic mean (UPGMA) and calculating the Dice correlation coefficient with a maximum position tolerance of 1% on the active zone (1% to 90%).

**(ii) MLVA.** The MLVA type was determined as previously described (12), for the inoculated strain and for 30 *Ye* isolates from pigs at 21 or 56 days after oral or nasal inoculation of pigs. Briefly, two multiplex PCRs (V2A, V4, V6, and V5, V7, V9), were run separately using an ABI 3130 DNA analyzer (Applied Biosystems, Foster City, CA, USA) with dye set (DS-30) fragment analysis chemistry, according to the manufacturer’s instructions. Electrophoretic patterns of isolates were analyzed and compared using BioNumerics 7.6 software (Applied Maths). The ROX-labeled Geneflo 625 DNA ladder (EurX, Gdańsk, Poland) was used as an internal size standard, and the electrophoretic patterns of the Y09AL405 strain were used as the reference patterns.

### Statistical analyses.

The influence of groups on weight or temperature was assessed each day using (i) a Kruskal–Wallis test when comparing the 3 groups of pigs (c, o, and n) and (ii) a Wilcoxon *t* test with the Holm adjustment for multiple pairwise group comparisons. The kruskal.test and pairwise.wilcox.test functions of R software were applied.

The influence of factors (group and/or trial period) on the proportion of positive swabs, feces or organs was assessed by a chi-squared test when considering the parameters separately (separate pigs, separate periods, and unpaired data). An exact Fisher correction was applied when at least one of the theoretical rates was lower than 5. Agreement of *Ye* detection in a pig considering the nature of samples, such as comparison between tonsil swab and feces samples or tonsil swabs and minced tonsil samples (paired data), was tested using McNemar’s chi-squared test. When the number of discordant pairs was lower than 10, the continuity correction was applied.

Data were analyzed with R software (v.3.5.2). Statistical significance was assigned when *P* values were lower than 0.05.
