# Supplementary material for: Controlled Experimental Infection in Pigs with a Strain of Yersinia enterocolitica Harboring Genetic Markers for Human Pathogenicity: Colonization and Stability
Source: Infect Immun. 2023 May 31;91(7):e00157-23. doi: 10.1128/iai.00157-23 (PMC10353419; doi:10.1128/iai.00157-23)
Supplement: Supplemental file 1 — Fig. S1 and S2 and Tables S1 and S2. Download iai.00157-23-s0001.pdf, PDF file, 0.4 MB [file iai.00157-23-s0001.pdf]

SUPPELMENTAL FIGURES AND TABLES

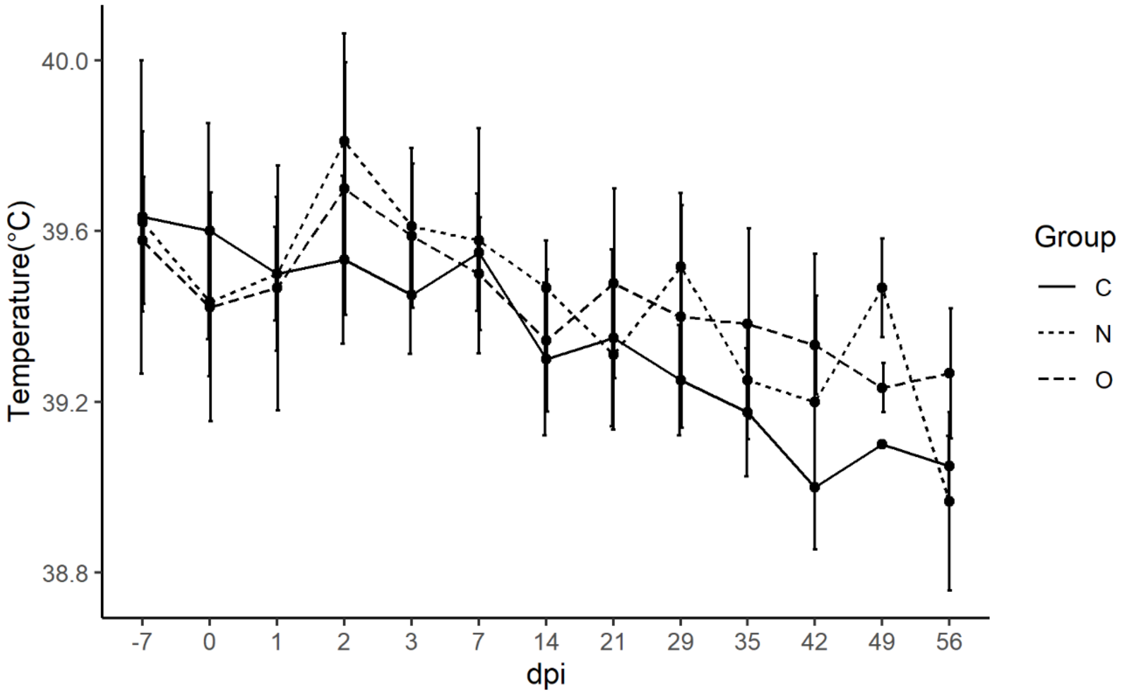

FIG S1 Mean rectal temperatures across the trial in control pigs (c), and orally (o) or nasally (n) inoculated groups. *: indicates 2 dpi, when pyrexia was observed for 33% (6/18) of the inoculated pigs.

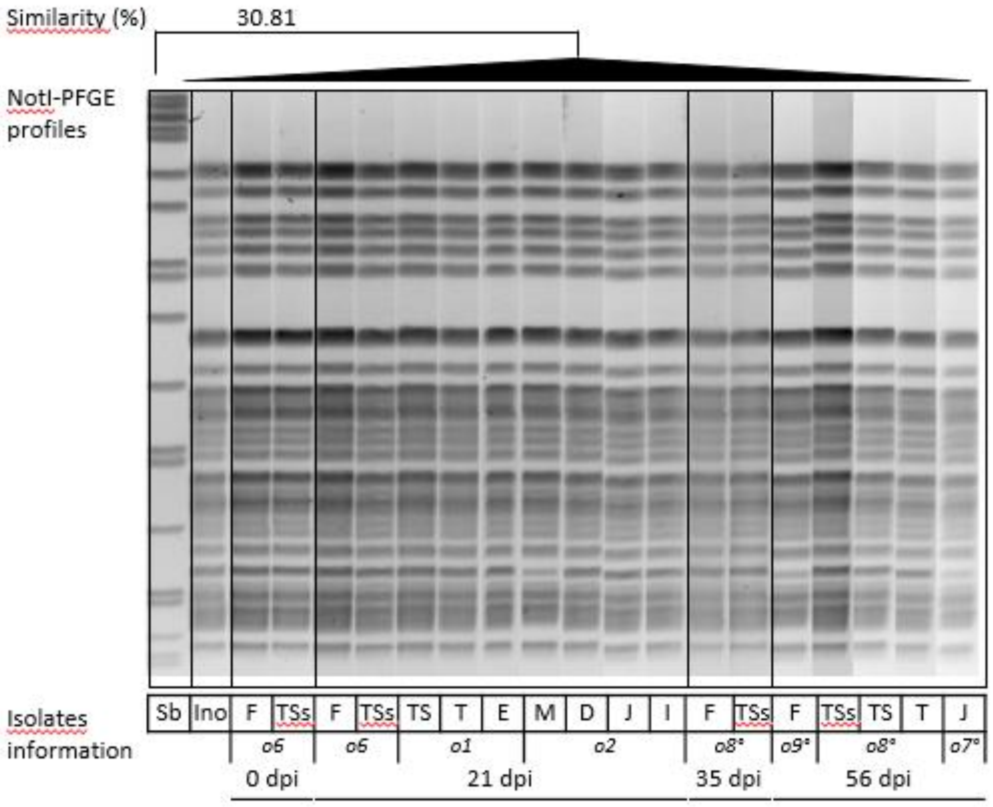

FIG S2 Dendrogram of NotI-PFGE profiles for *Y. enterocolitica* isolates recovered at different timepoints during the trial. PFGE profile of the inoculum (Ino) and of one isolate per *Ye*-positive sample type collected during the trial of pigs o1, o2, o6, o7°, o8° or o9°. Sample type: feces (F), tonsil swab (TSs), tongue (T), tonsil (TS), esophagus (E), mesenteric lymph nodes (M), spleen (S), liver (L) and intestinal content of duodenum (D), jejunum (J), ileum (I). Sb, *Salmonella* Braenderup H9812, size marker. dpi, day post-inoculation.

TABLE S1 List of microbiological agents in the specific pathogen-free status of pigs.

Agents	Name
**Viruses**	Classical and African swine fever
	Aujeszky's disease
	Foot-and-mouth disease
	H1N1/H3N2 swine influenza
	Transmissible gastroenteritis/porcine respiratory
	Porcine parvovirus
	Reproductive and respiratory syndrome virus
	Porcine circovirus type 2
	Border disease
**Bacteria**	*Mycoplasma hyopneumoniae*
	Pasteurella multocida
	Bordetella bronchiseptica
	Actinobacillus pleuropneumoniae
	Haemophilus parasuis
	Streptococcus suis Type 2
	Salmonella spp.
	Lawsonia intracellularis
	Brachyspira hyodysenteriae
	Yersinia spp.
	Campylobacter spp.
	Listeria monocytogenes
**Parasites**	*Balantidium coli*
	Trichomonas spp.

TABLE S2 The 23 isolates selected for NotI-PFGE analysis. PFGE profiles were determined for isolates coming from samples collected in pigs necropsied at 21 and 56 days post-inoculation (dpi). Samples originating from feces (F), tonsil swab (TsS), tonsil (Ts), tongue (T), esophagus (E), intestinal contents of duodenum (D), jejunum (J), ileum (I) and mesenteric lymph node (M).

Date of sampling	Sample	Pig reference
0 dpi	F	o6
	TsS	o1, o2, o6, o7°, o8°, o9
21 dpi	F	o6
	TsS	o6
	Ts	o1
	T	o1
	E	o1
	D	o2
	J	o2
	I	o2
	M	o2
35 dpi	F	o8°
	TsS	o8°
56 dpi	F	o9°
	TsS	o8°
	Ts	o8°
	T	o8°
	J	o7°
